# Diatom ecological response to deposition of the 833-850 CE White River Ash (east lobe) ashfall in a small subarctic Canadian lake

**DOI:** 10.7717/peerj.6269

**Published:** 2019-01-25

**Authors:** Scott J. Hutchinson, Paul B. Hamilton, R. Timothy Patterson, Jennifer M. Galloway, Nawaf A. Nasser, Christopher Spence, Hendrik Falck

**Affiliations:** 1 Ottawa-Carleton Geoscience Center and Department of Earth Sciences, Carleton University, Ottawa, Ontario, Canada; 2 Research Division, Canadian Museum of Nature, Ottawa, Ontario, Canada; 3 Geological Survey of Canada Calgary/ Commission Géologique du Canada, Calgary, Alberta, Canada; 4 Environment and Climate Change Canada, Saskatoon, Saskatchewan, Canada; 5 Northwest Territories Geological Survey, Yellowknife, Northwest Territories, Canada

**Keywords:** Aquatic ecosystems, Palaeolimnology, Diatoms, Tephra, Lake, Volcanic eruption

## Abstract

A <5 mm thick volcanic ashfall layer associated with the White River Ash (east lobe [WRAe]) originating from the eruption of Mount Churchill, Alaska (833-850 CE; 1,117–1,100 cal BP) was observed in two freeze cores obtained from Pocket Lake (62.5090°N, −114.3719°W), a small subarctic lake located within the city limits of Yellowknife, Northwest Territories, Canada. Here we analyze changes in diatom assemblages to assess impact of tephra deposition on the aquatic biota of a subarctic lake. In a well-dated core constrained by 8 radiocarbon dates, diatom counts were carried out at 1-mm intervals through an interval spanning  1 cm above and below the tephra layer with each 1 mm sub-sample represented about 2 years of deposition. Non-metric Multidimensional Scaling (NMDS) and Stratigraphically Constrained Incremental Sum of Squares (CONISS) analyses were carried out and three distinct diatom assemblages were identified throughout the interval. The lowermost “Pre-WRAe Assemblage (Pre-WRAeA)” was indicative of slightly acidic and eutrophic lacustrine conditions. Winter deposition of the tephra layer drove a subsequent diatom flora shift to the “WRAe Assemblage (WRAeA)” the following spring. The WRAeA contained elevated abundances of taxa associated with oligotrophic, nutrient depleted and slightly more alkaline lake waters. These changes were only apparent in samples within the WRAe containing interval indicating that they were short lived and only sustained for a single year of deposition. Immediately above the WRAe horizon, a third, “Post-WRAe Assemblage (Post-WRAeA)” was observed. This assemblage was initially similar to that of the Pre-WRAeA but gradually became more distinct upwards, likely due to climatic patterns independent of the WRAe event. These results suggest that lacustrine environments are sensitive to perturbations such as deposition of ash fall, but that ecological communities in subarctic systems can also have high resilience and can recover rapidly. If subsampling of the freeze cores was carried out at a more standard resolution (0.5–1 cm) these subtle diatom ecological responses to perturbation associated with the WRAe depositional event would not have been observed. This research illustrates the importance of high-resolution subsampling when studying the environmental impact of geologically “near instantaneous” events such as episodic deposition of ashfalls.

## Introduction

Plinian-style volcanic eruptions eject a massive amount of ash into the upper atmosphere. Although rare, these events can present a hazard over a large geographic area and have the potential to impact both societies and environmental systems (Walker & Croasdale, 1971). Significant input of ash into the atmosphere can also impact air quality and elicit changes in global climate (Robock & Mao, 1995). Lacustrine environments are particularly susceptible to such events as the deposition of tephra has the potential to radically alter water quality. For example, previous studies have found that depending on composition of the tephra and lake sediments, dissolution of the tephra can potentially result in lake acidification or a large increase in dissolved silica, which may alter a lake’s ecosystem and its ecosystem services (Telford et al., 2004). The introduction of ash into the water column of a lake can also impact water clarity and light penetration, which can impact biological productivity, including marcophyte growth (Abella, 1988; Urrutia et al., 2007).

Due to typically long intervals between Plinian eruptions, modern environmental monitoring is unlikely to be useful for accurately predicting the potential impact of episodic ashfalls (Tilling & Lipman, 1993). Therefore, data pertaining to their ecological impact must be obtained from available proxies. In northern Canada, lake sediments are well suited to fill this role due to their abundance and distribution, the diverse proxies they contain, and the near-continuous archives they provide. Variables related to regional environment and climate such as sedimentation rate, vegetation, precipitation, and bedrock composition influence water quality (e.g., pH, salinity, light penetration, nutrient concentration), which can be recorded in various proxies preserved in lake sediments (Battarbee, 2000). Diatoms preserved in lake sediments are particularly useful for reconstruction of past ecological change in northern North America due to their ubiquitous distribution, sensitivity to environmental change, and generally good preservation in lake sediments (Dixit et al., 1992). Diatoms make up a large proportion of the primary producers in most lakes and are a major contributor to carbon fixation (Smetacek, 1999). As a result, they play a key role in energy transfer and nutrient cycling between different trophic levels thereby strongly influencing the overall ecology in a lake (Brett & Muller-Navarra, 1997). Different taxa occupy narrow ecological optima and even small changes in water quality can elicit an observable response in the relative proportions of different species within a population. Differences in the diatom assemblages before and after deposition of tephra can indicate changes in the aquatic environment that resulted from tephra deposition (Dixit et al., 1992). Investigating the diatom response to these events also allows for a more comprehensive understanding of the factors controlling the distribution of different diatom species. This allows for the specific changes in populations to be linked to changes in the lacustrine environment allowing higher quality paleoenvironmental reconstructions to be developed leading to more accurate predictions of future changes and events.

A 3–5 mm thick layer of volcanic tephra was observed in two freeze cores collected in 2012 from Pocket Lake (62.5090°N, −.114.3719°W), a small subarctic lake located within the city limits of Yellowknife, Northwest Territories, Canada. This material was previously identified as part of the White River Ash Eastern Lobe (WRAe), which was deposited following the eruption of Mount Churchill, Alaska (1,117–1,100 cal BP; Patterson et al., 2017). The Pocket Lake deposit represents the only known occurrence of the WRAe in central NWT. The closest known WRAe deposit in a peatland setting is ∼100 km to the west and the nearest lacustrine accumulation is ∼470 km northwest (Robinson, 2001).

Although several studies have evaluated the algal ecological response to air fall tephra into lacustrine environments (Harper, Howorth & McLeod, 1986; Lotter et al., 1992; Lotter & Birks, 1993; Hickman & Reasoner, 1994; Hickman & Reasoner, 1998; Lotter et al., 1998; Eastwood et al., 2002; Barker et al., 2003; Telford et al., 2004; Cruces et al., 2006; Urrutia et al., 2007; Jovanovska et al., 2016) little data is available for the impact of episodic tephra deposition in lakes in subarctic regions. Ash layers reported previously in the literature are also generally at least an order of magnitude thicker than the one observed in Pocket Lake (Harper, Howorth & McLeod, 1986; Lotter & Birks, 1993; Lotter, Birks & Zolitschka, 1994; Hickman & Reasoner, 1994; Jovanovska et al., 2016). Ecological response of lacustrine systems to ashfall varies considerably, from no observed impact to considerable influence on diatom assemblages, making it difficult to identify causal relationships. The difference in response is likely related to the influence of various confounding variables (e.g., lake morphology, ash lithology, lake sediment composition, water chemistry, pre-existing lake ecology), all of which will influence the species makeup of the diatom assemblages (Lotter & Birks, 1993). The identification of a thin WRAe unit in two cores from Pocket Lake provides a unique opportunity to assess the ecological response of diatoms to the deposition of tephra in a small, subarctic lake. The primary objectives of this study are thus to:

• Evaluate at high temporal resolution (1 mm = ∼2 years) the ecological sensitivity and rate of recovery of diatom communities in Pocket Lake following perturbation of the lacustrine system by the WRAe ash fall;
• Compare the WRAe-driven ecological response of the diatom assemblage in Pocket Lake with previously published data from other lacustrine environments impacted by the deposition of ashfall tephra to better understand how confounding variables influence the response of primary producers in aquatic ecosystems and their impact on overall lake ecology.

### The Mount Churchill Eruptions and the White River Ash

The WRA depositional plumes were derived from two eruptions of Mount Churchill and distributed in two spatially distinct lobes. The older ca. 1,900 cal BP lobe extended north-south, parallel to the Yukon-Alaska border, while the younger WRAe lobe, dated at ca. 1,117–1,100 cal BP (833–850 CE), was much larger and extended east–west (Lerbekmo et al., 1975). The latter was the source of the material observed in Pocket Lake (Patterson et al., 2017). The WRAe eruption ejected tephra with a calculated volume of 47 km^3^ making it one of the largest known Plinian style eruptions during the Holocene (Lerbekmo, 2008). Deposits of the WRAe vary in thickness from 0.5 m to only a few millimeters at more distal locations (Robinson, 2001; Lerbekmo, 2008; Patterson et al., 2017).

The orientation of each WRA lobe was likely correlated with the prevailing seasonal wind patterns during each eruption (Hanson, 1965; West & Donaldson, 2002). Convincing evidence presented in several previous studies indicates that the eastward oriented WRAe eruption most likely occurred during late fall and winter, as winds in Alaska tend to trend eastward during winter (West & Donaldson, 2002). Winter emplacement of this tephra is also supported by the presence of anomalous steeply oriented deposits throughout the region (Hanson, 1965). Deposition of this type can only occur when concentrations of ash become compacted under snow, as rain during warmer seasons of the year would have quickly dispersed the ash (Lerbekmo & Campbell, 1968). Moreover, West (2007) analyzed the stratigraphy of two deposits of the WRAe along Bock’s Brook and Danjek River in the Yukon Territory where deposition occurred onto a floodplain and concluded that the eruption probably occurred in the late fall-early winter, just prior to the first snowfall but when seasonal temperatures remained consistently below freezing. At Bock’s Brook, the ash is preserved as a distinct layer overlain by a gravel unit with no visible reworking of sediment, suggesting that the ash froze in place immediately after deposition. The ash was subsequently buried during the following spring freshet when flood sediments buried and protected the WRAe deposits from being reworked. At Djenek River, West (2007) observed intact clasts of pumice embedded within the fluvial sediments deposited by the river, a type of deposition which requires frost in order to occur. As with the previous site, this material would have remained frozen until the spring. Spring flooding may have then caused the frozen ash layer to fracture, thereby causing the clasts to become embedded within the fluvial silt. The clasts remained intact, which indicated that they were frozen during fragmentation and transport, otherwise signs of abrasion would have been observed. Due to freezing temperatures and associated ice cover of the lake at the time of deposition, ash materials preserved in the Pocket Lake core were most likely delivered into the lake basin during two phases: the first occurring rapidly during the spring snow melt from the immediate lake area, and; the second being introduced gradually from watershed runoff during the following months.

## Study Site

**Figure 1:**
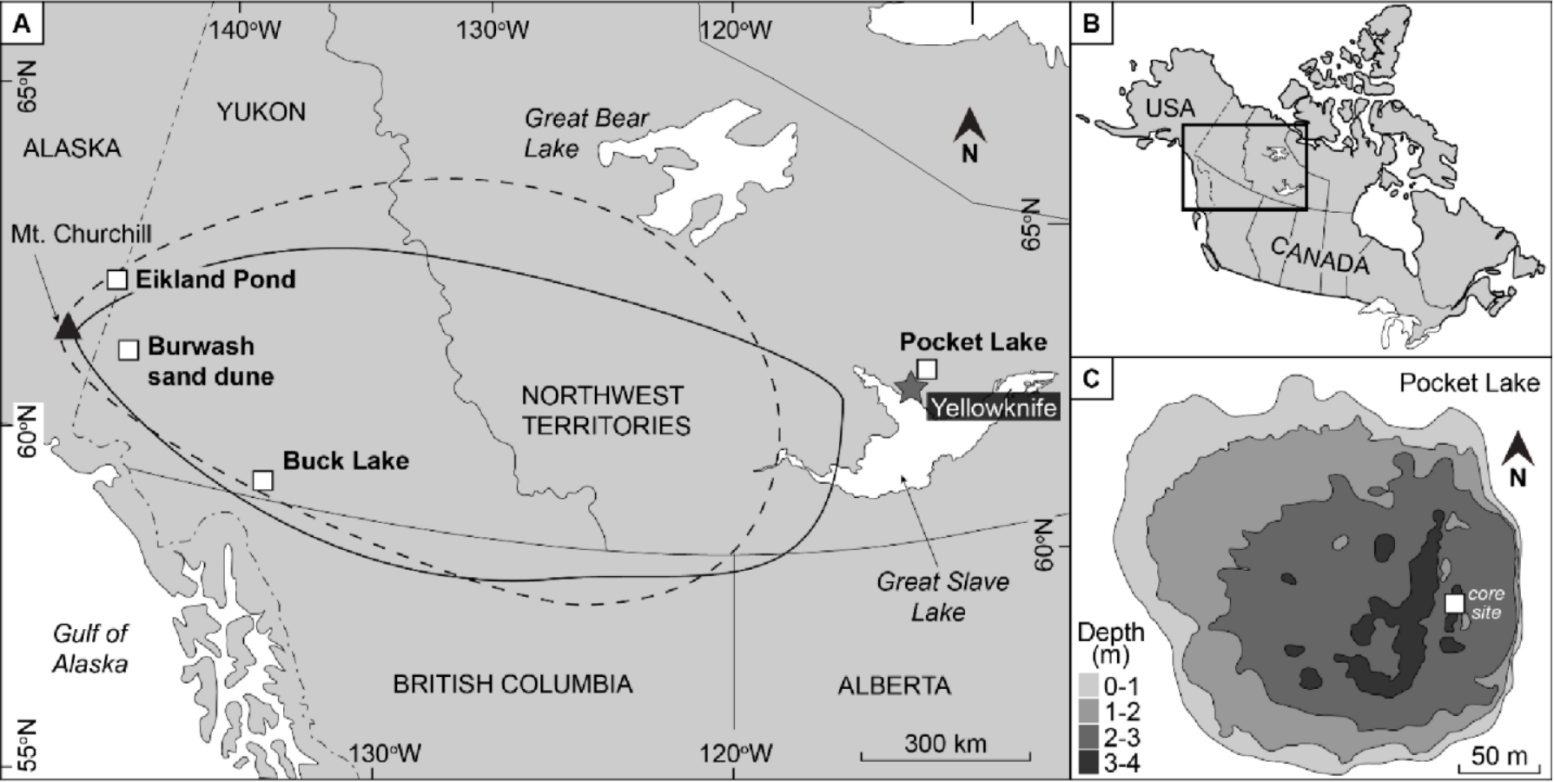
The location of the study site relative to Mount Churchill with the WRAe extent given by Lerbekmo (2008; dashed line) and by [Bibr ref-44][Bibr ref-44]; solid line). Eilkland Pond, Burwash sand dune, and Buck Lake indicate locations where the WRAe had previously been documented. The previously collected data from these two locations provided a reference with which the geochemical signal of the material in Pocket Lake was compared. Redrafted after Patterson et al. (2017).

Pocket Lake (62.5090 N, −114.3719 W) is a subarctic lake within the City of Yellowknife, Northwest Territories (NWT), Canada. It is a small headwater lake in the southern portion of the Baker Creek watershed. The catchment of Pocket Lake (Fig. 1) is less than 5 ha, with outcropping bedrock that drains south into the lake via a soil-filled valley (Spence, 2006). A recent study identified a layer of tephra in two cores collected from Pocket Lake (Patterson et al., 2017). The layer is composed of white-coloured ash forming a band 3–5 mm thick. Based on ^14^C-derived age models, stratigraphy, shard morphology and the geochemical signature of the material, obtained through wavelength dispersive X-ray analysis at Carleton University, the tephra layer was identified to be the White River Ash Eastern Lobe (WRAe). Cryptotephra associated with the WRAe have been recorded in lakes in Newfoundland, Greenland, and even as far east as Poland (Jensen et al., 2014; Watson et al., 2016a; Watson et al., 2016b). The occurrence of the WRAe in Pocket Lake represents the easternmost recorded macroscopic deposit and is the first reported occurrence of the WRAe in a lacustrine environment from the central NWT (Patterson et al., 2017).

## Methods

Two cores, approximately 3 m apart, were collected from Pocket Lake during winter 2012 and provided the material used in this study (Fig. 1). The cores, named PKT_1FR and PKT_2FR1, were composed primarily of massive gyttja-type mud. They were collected using a freeze corer, which uses dry ice to freeze to sediment cores *in situ* to reduce the risk of homogenization of soupy sediment-water interface material and permitted extremely high resolution sub-sampling (Galloway et al., 2010; Macumber et al., 2011; Macumber et al., 2018; Dalton et al., 2018). Sub-sampling of cores PKT_1FR and PKT_2FR1 was conducted using a custom sledge microtome (Macumber et al., 2011) at intervals of 1 mm to 1 cm throughout their lengths. The first core (PKT_2FR1) was 131 cm long, while the second core (PKT_1FR) was 180 cm long. The sediment-water interface was not captured in core PKT_1FR; core PKT_2FR1 was therefore used for detailed study. The tephra appeared as a thin band of white material spanning 55.4–55.7 cm depth and was visible upon coring. The intact cores were inspected under X-ray and the images generated did not indicate any substantial bioturbation/mixing throughout the interval used here. The contact between the WRAe and the surrounding sediments was sharp and unbroken. Contiguous 1 mm sub-samples from 54.5 to 56.4 cm spanning an interval from 1 cm above to 1 cm below the tephra layer in core PKT_2FR1 were prepared for diatom analysis.

Sub-sampled material was placed in individual centrifuge tubes and freeze dried. Aliquots of 10 mg from each tube were placed in individual beakers. Then 10 ml of concentrated sulfuric (H_2_SO_4(aq)_) and nitric (HNO_3(aq)_) acid solution, containing both in equal proportions was added and heated to digest organic material. Following digestion, the acid was diluted and removed through centrifugation dilution, and this was repeated five times. A 0.8 ml subsample slurry was then placed using a volumetric pipette onto clear coverglass and left to dry for 24 h. The coverglass subsamples were mounted to glass slides using Naphrax^®^ (Brunel Microscopes, Chippenham, UK), a permanent adhesive with a refractive index of 1.73.

**Figure 2:**
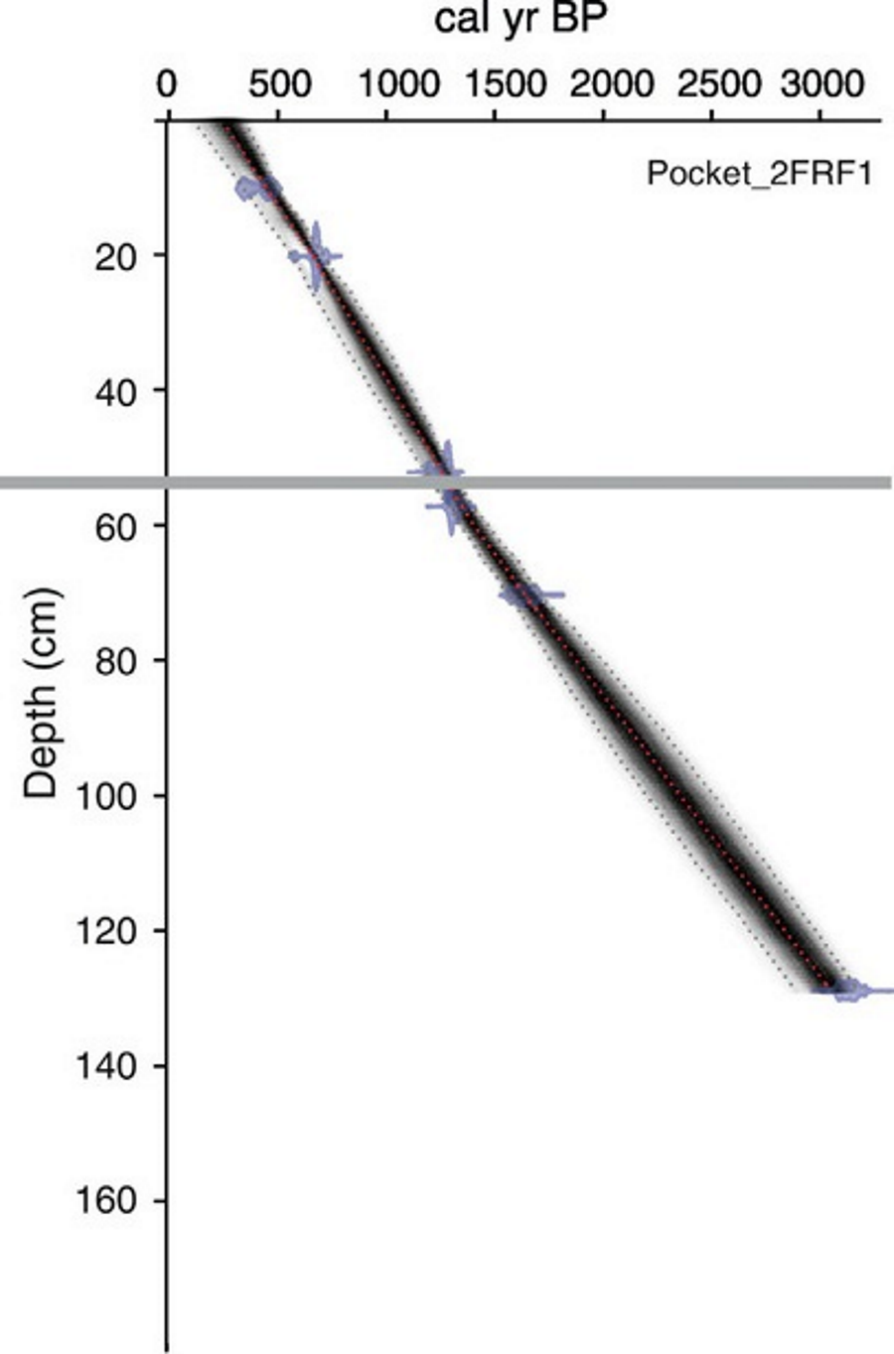
Age-depth model produced for PKT_2FR1. The grey line delineates the depth of the WRAe (1,110 ± 50 cal BP). Redrafted after Patterson et al. (2017).

An age-depth model was generated for both cores based on radiocarbon dates obtained from bulk sediments at eight horizons throughout the 131 cm long PKT_2FR1 core (Fig. 2; Table 1; Patterson et al., 2017). Two of these horizons were located immediately above and below the interval of interest (57 cm and 52 cm). Dates from these locations along with the previously documented time of deposition for the WRAe allowed for a highly accurate age-depth model throughout the study interval (56.4–54.5 cm). Samples were pretreated with HCl to remove carbonates. Radiocarbon dates were obtained using the accelerator mass spectrometer at the 14CHRONO Dating Laboratory in Belfast, United Kingdom. AMS radiocarbon dates were calibrated with OxCal v4.2 (Bronk Ramsey, 2009) and the IntCal13 calibration curve (Reimer et al., 2013). Age-depth relationships for the cores were developed using Bacon 2.2 (Blaauw & Christen, 2011; Blaauw & Christen, 2013) applying accumulation rate and memory parameter values based on lakes in the central NWT (Crann et al., 2015).

**Table 1:** Radiocarbon dates from PKT_2FR1. Dates were calibrated with the intCal13 calibration curve (Reimer et al., 2013) using OxCal v4.2.4 following the methods given in Millard (2014). The tephra date is based on wiggle-match date for the WRAe (Jensen et al., 2014). Redrafted after Patterson et al. (2017).

	**Lab ID**	**Depth (cm)**	**14C age BP ± 1 *σ***	**Pretreatment**	**Cal BP ± 2 *σ***
**PKT_2FR1**	UBA-20676	10–10.5	362 ± 27	Acid Only	500–422 (50.7%)
					400–316 (44.7%)
	UBA-22350	20–20.5	731 ± 31	Acid Only	727–653 (95.4%)
	UBA-20679	52–52.5	1335 ± 25	Acid Only	1,302–1,239 (85.5%)
					1,205–1,186 (9.9%)
	**Tephra**	**55.4–55.7**			**1,110 ± 50**
	UBA-22351	57–57.5	1,394 ± 30	Acid Only	1,350–1,279 (95.4%)
	UBA-22352	70–70.5	1,725 ± 31	Acid Only	1,707–1,561 (95.4%)
	UBA-20678	128.5–129	2,966 ± 26	Acid Only	3,215–3,057 (93.9%)

Diatom counts were made using an Olympus BX51 light microscope at 1000x under oil immersion with a minimum of 600 valves enumerated per sample. Counts were collected from 20 samples at 1-mm intervals between 54.5 and 56.4 cm depth. Diatom taxa were identified at the lowest possible taxonomic level with reference to photomicrographs of taxa from similar geographic regions and environmental conditions (Krammer & Lange-Bertalot, 1985–1991; Hartley, 1996). Species names were corrected to current taxonomic nomenclature following Algaebase (Guiry & Guiry, 2018). Diatom counts were expressed in relative abundance and plotted stratigraphically using C2 version 1.7 (Juggins, 2014).

Significant taxa were identified using the methodology outlined in Patterson & Fishbein (1989). Only taxa present in significant numbers in at least one sample throughout the interval were included in subsequent analyses. To determine this, the standard error was calculated for the individual taxon in each subsample. If this value exceeded the abundance of a taxon, it was deemed insignificant in that sample (Patterson & Fishbein, 1989). Of the 79 taxa identified throughout the interval only 38 were found to be significant enough to be included. Non-metric multidimensional Scaling (NMDS) analysis was used to ordinate samples based on similar diatom taxa to visualize changes at the assemblage scale (*n* = 20 samples). NMDS allowed visually grouping of samples containing similar assemblages as well as the taxa that defined each group. It was chosen over other multivariant ordination techniques because it allowed for samples to be assessed based solely on similarities in assemblage structure without assuming any underlying variable-environmental gradient relationships (Paliy & Shankar, 2016). In addition, Stratigraphically Constrained Incremental Sum of Squares (CONISS) cluster analysis was conducted using the rioja package in RStudio to identify stratigraphic zones based on changes in the diatom community composition. Visual inspection coupled with the results provided via CONISS and NMDS were used to define stratigraphic zones based on diatom assemblages.

## Results

**Figure 3:**
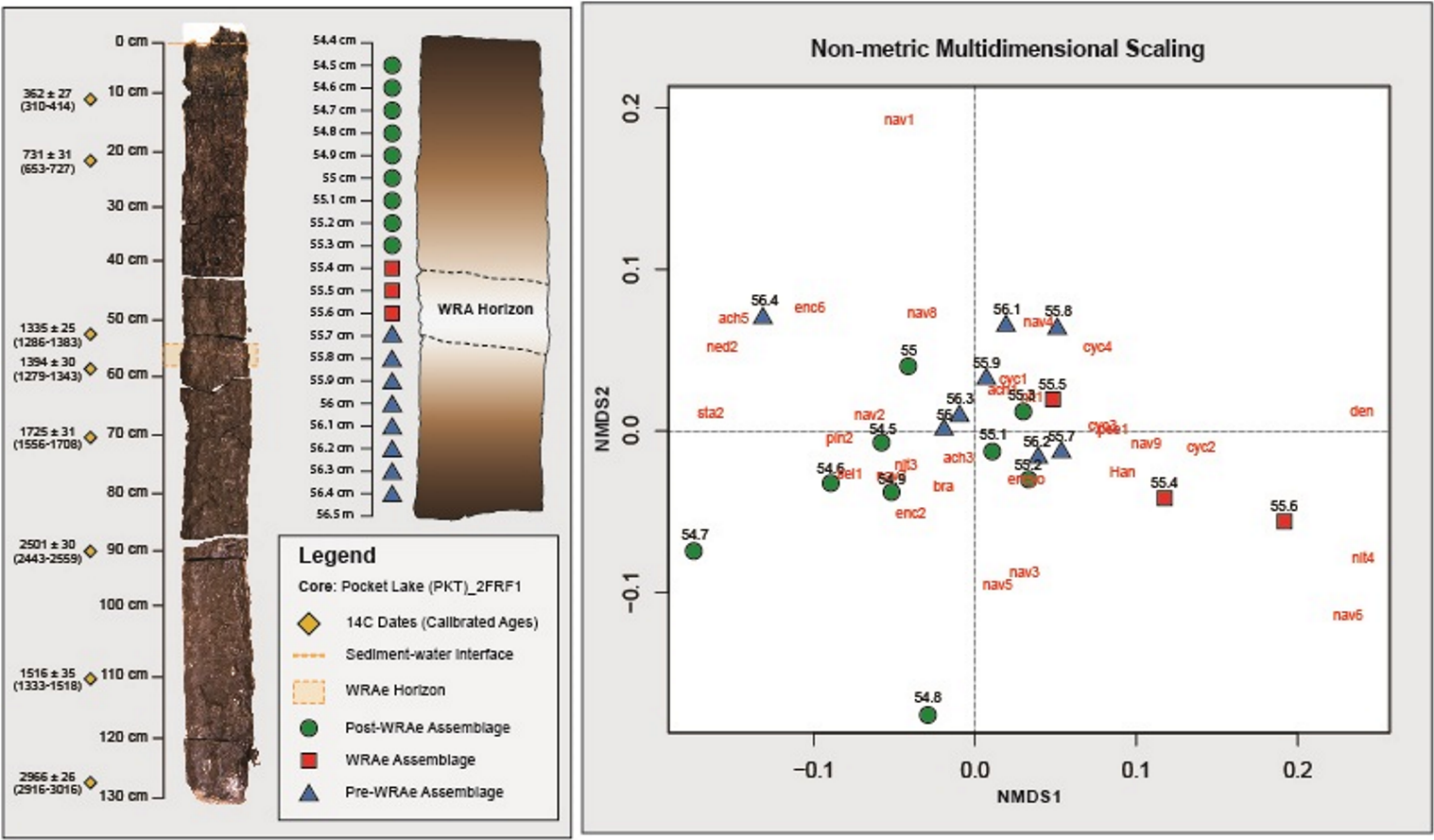
Non-metric Multidimensional Scaling analysis. Samples containing a similar diatom composition are grouped. Grouping is observed between the Pre-WRAe Assemblage (green), the WRAe Associated Assemblage (red), and the Post-WRAe Assemblage (blue). Stratigraphic location of each sample in the core is indicated on the left panel and the radiocarbon dates used to develop the age-depth model are plotted beside the photographed core. Dotted lines illustrate the top and bottom of the WRAe horizon. The first three letters of each taxon represents the genus name and each numeric value indicates a unique species within the genera. Where only the genus name is given, only one member was recorded. Nav, *Navicula*; Ency, *Encyonema*; Sell, *Sellaphora*; Pseud, *Pseudostaurosira*; Psam, *Psamnothidium*; Cyc, *Cyclotella* sensu lato; Den, *Denticula*; Bra, *Brachysira*; Ach, *Achnanthidium*; Ned, *Neidium*; Cym, *Cymbopleura*; Staur, *Stauroneis*; Pinn, *Pinnularia*; Han, *Hantzschia*.

The tephra in core PKT_2FR1 appeared as a 3–5 mm white band composed of clear, glassy shards at 55.4–55.7 cm depth (Patterson et al., 2017; Fig. 3). Based on the age-depth model generated for this core, the core captured sediments as old as 3,215–3,057 cal yr BP. The WRAe horizon, deposited 1,117–1,100 cal BP, occurred at 55.4 cm depth, with a modelled sedimentation rate of ∼2 yrs/mm (Patterson et al., 2017). Due to the timing and nature of the Mount Churchill eruption, the addition of the tephra would have led to the amount of material deposited that year being much greater than normal. Consequently, the four samples containing the ash layer (55.4–55.7 cm) collectively represented a single season of deposition (1 year). The well-defined age of the tephra layer as well as the two radiocarbon dates obtained above and below the interval of interest (52 and 57 cm depth; Fig. 2; Table 1) allowed for a high degree of confidence in the calculated time represented in each sample.

Seventy-nine diatom taxa were identified in the 20 samples examined through the 2 cm studied interval, which included the WRAe tephra, and were dominated by benthic taxa. Planktic (centric) diatoms such as *Discostella pseudostelligera*, *Cyclotella distinguenda*, and *Lindavia michiganiana* were recorded, but together never exceeded 10% relative abundance. Above 55.9 cm, small centric taxa consistently decreased in relative abundance, almost disappearing above 54.8 cm depth.

**Figure 4:**
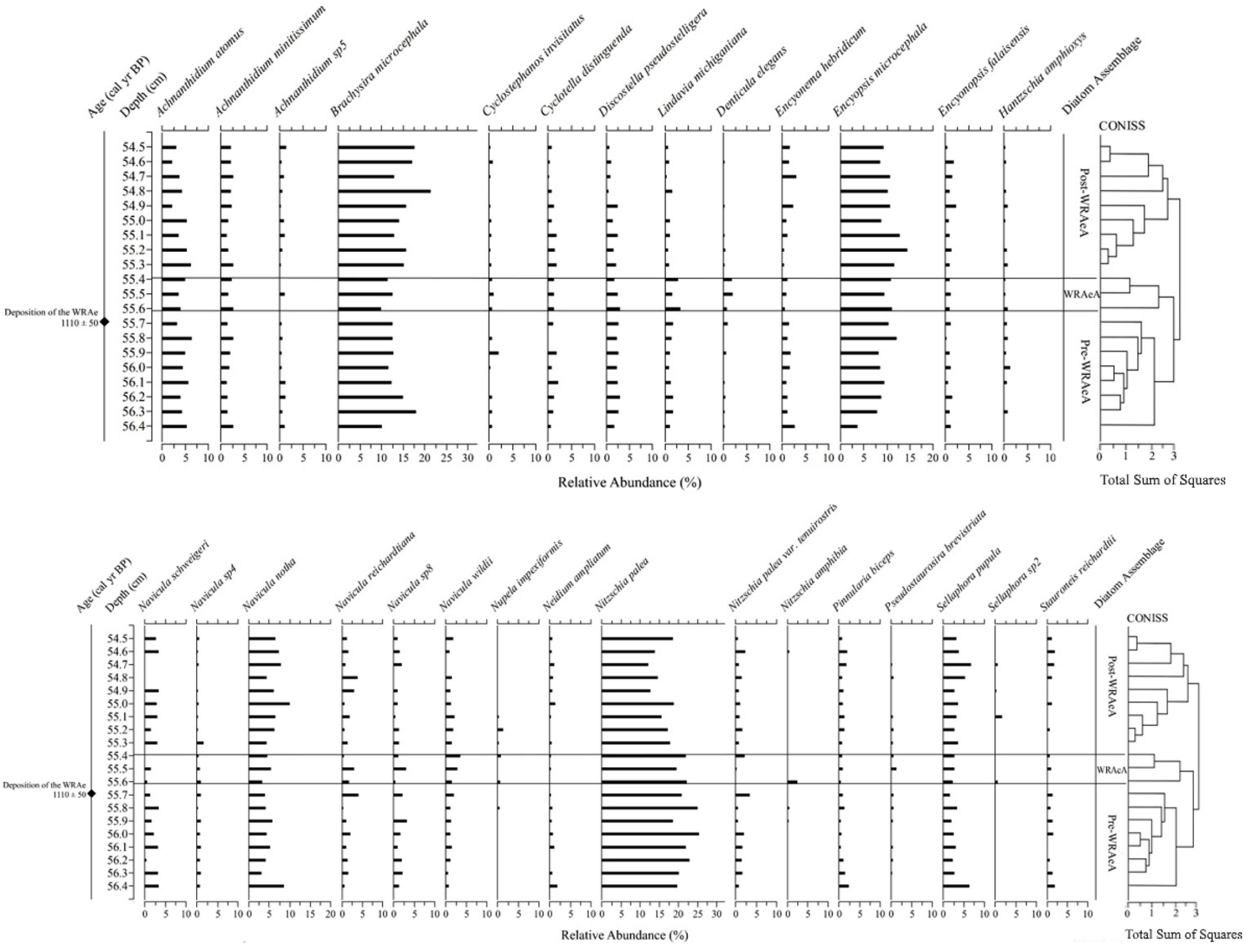
Relative abundance profiles of diatom taxa from Pocket Lake. Taxa are grouped based on morphology. Only taxa deemed statistically significant in at least one sample are included.

Based on the interpretations generated via CONISS and NMDS, three distinct diatom assemblages were identified in the 2 cm interval of the core: (1) the “Pre-WRAe Assemblage (Pre-WRAeA)” (56.4–55.7 cm); (2) the “WRAe Assemblage (WRAeA)” (55.6–55.4 cm); and the (3) the overlying “Post-WRAe Assemblage (Post-WRAeA)” (55.3–54.5 cm). Plotted changes in relative abundances of diatom taxa for the interval are used to illustrate these stratigraphic and temporal trends (Fig. 4).

The lowermost Pre-WRAeA is indicative of acidic and eutrophic conditions. In the Pre-WRAeA (56.4 to 55.9 cm, *n* = 6), *Nitzschia palea* (*sensu lato*) was the most common single species with a median abundance of 21.0% (min: 18.7%, max: 25.3%). *Navicula* species had a median abundance of 23.0% (min: 19.6%, max: 27.2%); the most common species were *Navicula schweigeri* (2.8%), *Navicula notha* (4.9%) and *Navicula cryptotenella* (7.2%). Important taxa of lesser abundance throughout this interval were *Brachysira microcephala* (12.6%), *Encyonopsis microcephala* (8.5%), *Discostella pseudostelligera* (2.5%), *Achnanthidium atomus* (4.8%), and *Achnanthidium minutissimum* (1.8%; Fig. 4). No significant trends were observed in the assemblage present throughout this interval.

A shift to the WRAeA occurred immediately following the deposition event (55.6 to 55.4 cm inclusive; *n* = 3). This interval contains material collectively representing one year of deposition. Although the 55.7 cm sample contained the tephra contact, the WRAeA was not observed. This could potentially be due to contamination from the underlying lake sediment causing the shift to be obscured. Again, this assemblage was dominated by *N. palea* (19.6%) and *Navicula* spp. (23.1%). The less abundant *Navicula wildii* was elevated in relative abundance between 55.6 and 55.4 cm depth, increasing from a median of 1.1% to 2.4% with a peak of 3.7% (55.4 cm). This increase was not observed in other *Navicula* species. A decrease in relative abundance was recorded for *B. microcephala, E. microcephala* as well as *A. atomus* and *A. minutissimum* for the same interval (Fig. 4). The relative abundance of centric diatom *L. michiganiana* was elevated throughout the tephra interval peaking twice at 55.6 and 55.4 cm depth, then followed by a sharp decline. Minor constituents of the WRAeA showed greater variability than the primary taxa. The relative abundance of *Denticula elegans* increased from a median value of ∼0.3% before the tephra to a maximum of 2.1% in this interval. Slight increases in relative abundances were also recorded for *N. amphibia* and *Pseudostaurosira brevistriata*. Conversely, a decrease in abundance was recorded for *Encyonema minutum* and *Hantzschia amphioxys, Encyonema hebridicum*, *N. schweigeri* and *N. ampliatum*.

Above 55.4 the Post-WRAeA was observed. The Post-WRAeA was initially similar to the Pre-WRAeA but became more unique upwards. Between 55.1 cm and 54.5 cm, the relative abundance of centric diatoms consistently decreased from 6.1% to 2.6%. *Nitzschia palea* initially decreased from 17.3% to 12.3% between 55.2 cm and 54.7 cm but subsequently recovered to a relative abundance of 18.6%. The opposite pattern was observed in *N. cryptotenella*, which increased in abundance over the same interval from 6.3% to 11.3%. Again, this was followed by a decline to 8.0%. Similar patterns were observed in the abundance of *B. microcephala and S. pupula*. For each taxa, a gradual increase was observed from ∼55.2 cm to ∼54.7 cm, increasing from 15.7% to 21.5% and 2.9 to 6.9 respectively.

## Discussion

The samples throughout the 54.5–56.4 cm depth interval were consistently dominated by benthic diatom taxa. Pocket Lake is 6 m deep at its—*Z*_*max*_ and has a surface area of 4 ha (Spence, 2000; Reid & Faria, 2004). The high proportion of benthic taxa may be related to the northern location and shallow depth of Pocket Lake. Shallower lakes allow more light penetration to the bottom of the lake resulting in the success of benthic taxa. Long periods of seasonal ice cover present in lakes in cold, northern climates limit light penetration, nutrient supply and the planktic zone for a significant part of the year leading to a diatom composition dominated by tychoplanktic and benthic taxa that are competitive in the moat surrounding retreating ice in spring (Smol, 1988).

Three distinct diatom assemblages characterized the 2 cm interval of the core: the lowermost Pre-WRAeA, the overlying WRAeA and the uppermost Post-WRAeA characterized by a partial return to pre-WRAe conditions. The lowermost Pre-WRAeA (56.4–55.7 cm) is indicative of slightly acidic and eutrophic conditions. *Nitzschia palea* and *B. microcephala* are tolerant of both acidic conditions and nutrient enrichment (Lange-Bertalot, 1979; Hamilton et al., 2015). The most common *Navicula* species in the Pocket Lake core are *N. schweigeri* and *N. cryptotenella*, which are moderately tolerant to pollution and are often associated with waters rich in organic material (Lange-Bertalot, 1979). Results from NMDS analysis show overlap between the upper samples of the Pre-WRAeA and the lowermost samples of the Post-WRAeA. This potentially indicates that the changes this assemblage documents was likely primarily derived from broad regional environmental trends independent from the Mount Churchill eruption. That is, the WRAe deposition was only a driving force in the first assemblage change, to the WRAeA and not the second shift. Hydrologic changes in Pocket Lake resulting from the WRAe were only sustained for the growing season immediately following the Mount Churchill event. Changes observed in the samples following the WRAe interval were therefore largely due to other broader environmental factors and not the Mount Churchill event. Taxa that dominated the Pre-WRAeA (e.g., *N. palea* and various *Navicula* spp) continued to be the most common taxa in the Post-WRAeA. However, gradual changes in the relative abundances of several other taxa (e.g., *B. microcephala*, several *Navicula* species*, S. reichardtii, N. ampliatum*) continued stratigraphically upwards through samples comprising the Post-WRAeA. Higher abundances of small planktic centric diatoms is often associated with warmer climates. Their observed decline in the Post-WRAeA potentially indicates that the region experienced a cooling climate (Lotter et al., 1998; Winder, Reuter & Schladow, 2009; Saros & Anderson, 2015).

The WRAeA (55.6–55.4 cm), which developed immediately after WRAe deposition, was characterized by a higher relative abundance of taxa associated with decreased productivity and more alkaline waters. This is most apparent in the relative abundances of *Navicula wildii* (1.2% to a peak of 3.7%), *Lindavia michiganiana* (1.2% to a peak of 3.5%), *Pseudostaurosira brevistriata* (∼0.3% to a peak of 0.8%) and *Denticula elegans* (<1% to a peak of 2.1%). These species indicate a slight shift away from the moderately eutrophic conditions inferred for Pocket Lake prior to deposition of the ash (Lange-Bertalot, 2001; Le Blanc, Gajewski & Hamilton, 2004). Increases in the abundance of *D. elegans* suggest greater sedimentation rates as the ash eroded into the lake from the catchment (Le Blanc, Gajewski & Hamilton, 2004). Tephra material added to a shallow lake like Pocket Lake may have led to increased turbidity that limited light penetration (Hickman & Reasoner, 1994). Additionally, tephra material covering the lakebed may have inhibited nutrient diffusion from the normal lake sediments into the overlying water column causing depletion in dissolved concentrations of certain nutrients (Hickman & Reasoner, 1998). Both mechanisms may have collectively reduced the overall productivity in the lake leading to the increase in oligotrophic taxa and reduction of taxa tolerant of eutrophic, and acidic, conditions. The relative abundance of several *Achnanthidium* species, particularly *A. minutissimum,* along with *B. microcephala*, decreased coincident within the tephra containing interval. Competition from other species favoured by the moderate changes in water chemistry might also be a contributing factor to the decrease in relative abundances (Lotter et al., 1998). However, in the Lotter et al. (1998) study, dissolution of tephra shards did not influence observed changes in diatom composition. Geochemical analysis of the tephra shards by Patterson et al. (2017), found them to be primarily rhyodacitic, with the dissolution rate being closely tied to the silica content present in the shard. Experimental studies have found that such silica-rich material will still require thousands of years to dissolve, even under slightly acidic conditions, and would thus not impact the water chemistry of the lake (Wolff-Boenisch, Gislason & Oelkers, 2004; Wolff-Boenisch et al., 2004). Other research has found that bacterial communities in natural water bodies can mediate the dissolution process of silica-rich tephra, substantially increasing the rate of dissolution but still not to the degree required to alter the water chemistry of Pocket Lake within the short time interval where changes in diatom composition are observed (Thorseth, Furnes & Tumyr, 1995; Staudigel et al., 1998; Brehm, Gorbushima & Mottershead, 2004).

The observed diatom assemblage response to the WRAe deposition was most apparent in samples containing tephra material, indicating that major changes in water property characteristics were only sustained during one season of deposition. The winter eruption of Mount Churchill resulted in the bulk of the volcanic ash falling out onto snow cover, which entered Pocket Lake the following spring carried in snow melt runoff from the catchment (Robinson, 2001; Lerbekmo, 2008; Patterson et al., 2017). Therefore, the samples containing the WRAe (55.4–55.7 cm inclusive; *n* = 4) material likely represent the same year. Results from both CONISS and NMDS indicated a shift to a new assemblage in samples contained within this interval. The samples above the interval form a third distinct group indicating the WRAe was not a significant factor controlling diatom assemblage beyond the year in which it was deposited. Although results from both CONISS and NMDS support the assemblages above and below the WRAe interval being distinct groups, plotted results from NMDS analysis show some overlap between them. Overlap between these two groups indicates that the diatom composition present in each was very similar. A shift back to an assemblage close in structure to the one preceding the WRAe suggests that at least a partial return to pre-WRAe conditions occurred the following year with the tephra no longer having a significant influence on hydrologic conditions within the lake.

Similar changes have been observed in several previous studies (e.g., Lotter & Birks (1993), ∼10 mm thick layer in two ancient lake beds; Birks & Lotter (1994), 78-mm thick layer, Holzmaar, Germany; Jovanovska et al. (2016), 15 cm thick in cores from Lakes Ohrid and Prespa). Other studies have reported no clear relationship between tephra layer thickness and impact on the diatom community (e.g., Hickman & Reasoner, 1994; Hickman & Reasoner, 1998, in studies on = ∼10 cm thick and ∼5 cm thick tephra layers). As discussed below sampling resolution may have influenced the ability to detect temporary ecological responses to tephra deposition.

The recovery rate of diatom populations reported in previous studies of lakes impacted by ash falls, and where the ash has impacted lake ecology, varies substantially. For example, one study documented gradual recovery of diatom populations over hundreds of years (Hickman & Reasoner, 1994), another reported a much shorter, 2–20-year recovery period (Hickman & Reasoner, 1998), and in Germany short-lived impacts of tephra deposition on lacustrine diatom communities ranging from ∼5–20 years were observed in the Black Forest region (Lotter & Birks, 1993). In other examples, Hickman & Reasoner (1994); Hickman & Reasoner (1998) assessed the impact of tephra units on the diatom flora in alpine lakes from Yoho and Banff national parks, Alberta, Canada, and found little change in the composition of the diatom community following tephra deposition aside from a slight increase in diversity. Similar to the transition from the Pre-WRAeA to the Post-WRAeA, Hickman & Reasoner (1998) attributed their observed changes in the relative abundance of diatoms to coeval changes in other environmental variables (e.g., precipitation, temperature, changes in vegetation) unrelated to tephra deposition.

Based on the varying responses of diatom assemblages to tephra deposition described above it is obvious that a variety of environmental factors influence diatom ecological responses to tephra deposition including lake morphology, water chemistry, lake sedimentology and the volume and thickness of tephra deposited (Lotter & Birks, 1993; Telford et al., 2004). In deeper lakes with established populations of planktic diatoms, the deposition of an ash layer appears to primarily impact benthic diatoms, leading to an increase in the relative abundance of planktic taxa (Lotter & Birks, 1993; Jovanovska et al., 2016). In shallow lakes, or lakes in regions with long periods of ice cover, a significant population of planktic diatoms cannot develop, and this pattern will not be observed (Wetzel, 1983; Rühland & Smol, 2005). Although many studies reported some response to the deposition of tephra, the significance of the relationship isn’t clear. Lotter & Birks (1993) used partial RDA analysis to determine differences in the response of diatoms to the Laacher See Tephra in two different sampling sites in the Black Forest, Southern Germany. They found that in their cases the tephra as well as the lake sedimentology contributed to the variance that was observed, but it was unclear which was most important as both variables interacted with each other. Observed changes in sedimentology could have resulted from the deposition of the tephra but may also be explained by unrelated phenomena.

Observation of the brief temporal response and subsequent rapid recovery of the diatom community to deposition of the WRAe in Pocket Lake may in part be a result of the high resolution of sub-sampling carried out in this study, which contrasts with the coarser temporal resolution analyses carried out on similar lakes where no diatom response to tephra deposition was recorded (Hickman & Reasoner, 1994; Hickman & Reasoner, 1998). It’s possible that the coarse sample resulted in the diatom tephra response to obscured and therefore not observed. In Pocket Lake, major changes in the proportion of different taxa only persisted for approximately one year. The importance of carrying out high-resolution sub-sampling of cores to test diatom ecological response may also be evident in the Lotter & Birks (1993) study on the impact of the Lascher See Tephra in Holzmaar, Germany. There the impact of the tephra deposition on the environment was also short lived, with recovery occurring in 10–20 years, but due to the sampling resolution employed these changes could only be observed at decadal resolution (Lotter & Birks, 1993).

## Conclusions

The results of the ecological impact of WRAe tephra deposition in Pocket Lake show that there were short-duration changes in diatom flora. NMDS coupled with CONISS analysis reveal three distinct assemblages throughout the 2.0 cm interval, which included a 3–5 mm thick tephra layer; the “Pre-WRAe Assemblage (Pre-WRAeA)”, the “WRAe Assemblage (WRAeA)”, and the “Post-WRAe Assemblage (Post-WRAeA)”. In the basal Pre-WRAeA, the observed taxa are indicative of slightly acidic and eutrophic lake conditions. The WRAeA is associated with deposition of the WRAe, and is characterized by taxa associated with nutrient depletion and oligotrophic and mildly alkaline conditions. The WRAeA also suggests a change in lake conditions associated with deposition of the volcanic ash that resulted in a decrease in overall productivity, reduced water clarity and a slow down in nutrient exchange with lake bottom sediments. This shift was only sustained for a single year following ash deposition, after which the lake diatom community partially recovered. Immediately above the WRAe interval, a shift to the Post-WRAeA occurs. This assemblage is initially similar in structure to the Pre-WRAeA but moving up within the interval, this assemblage becomes gradually more unique showing an increase in taxa associated with decreased acidity and a colder climate. These trends are likely driven by changes in water quality associated with broader regional climatic trends. The results suggest that diatom communities in Pocket Lake were sensitive to deposition of the WRAe. Once the tephra was no longer being eroded from the watershed and the hydrology of the lake was able to partially recover to its initial conditions, the diatoms community rapidly recovered and sustained no long-term impact related to the WRAe depositional event. This was in part due to the very slow dissolution of the rhyodacitic tephra, which was essentially inert once deposited on the lake bottom. Due to the short-lived nature of the ecological shift, a mm-scale high-resolution subsampling strategy was necessary in order for the changes in the diatom assemblage to be recognized. In previous lakes studies where little to no response to an ash fall was observed, it is possible that similar short-lived changes in composition occurred but were not observed because the sub-sampling approach employed was too coarse. These findings emphasize the necessity of high-resolution sub-sampling strategies for paleolimnological studies attempting to recognize changes that might be relatively short lived or those that result from rare, instantaneous events.

##  Supplemental Information

10.7717/peerj.6269/supp-1 Figure S1 PKT_2FR1 core card Image of the core alongside X-ray and inverted X-ray images. The right panel indicates the location, depth, and eco-zone from which the core was obtained.

10.7717/peerj.6269/supp-2 Table S1 Diatom counts (Valves/sample) for each sample in the interval of interest

## Additional Information and Declarations

### Field Study Permissions

The following information was supplied relating to field study approvals (i.e., approving body and any reference numbers): Approval of field work was given by the Aurora Research Institute (14435 and 14965).

### Data Availability

The following information was supplied regarding data availability: Raw data is provided in the Supplementary Materials.
