# Supplementary figures and images for: Diatom ecological response to deposition of the 833-850 CE White River Ash (east lobe) ashfall in a small subarctic Canadian lake
Source: PeerJ. 2019 Jan 25;7:e6269. doi: 10.7717/peerj.6269 (PMC6348948; doi:10.7717/peerj.6269)

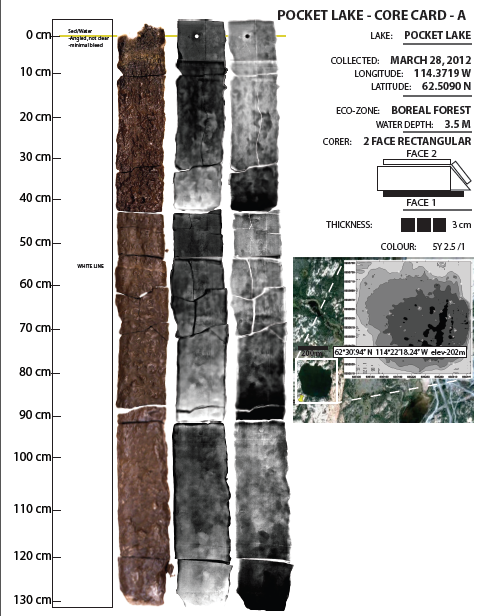

Supplement: Figure S1 — Image of the core alongside X-ray and inverted X-ray images. The right panel indicates the location, depth, and eco-zone from which the core was obtained. [file peerj-07-6269-s001.png]
